# Clinical decision-making in relation to autoimmunity: insights from catatonia and autoimmune encephalitis

**DOI:** 10.1192/bjo.2025.10951

**Published:** 2026-01-20

**Authors:** Almila Erol

**Affiliations:** Independent scholar, Minnesota, USA

**Keywords:** Autoimmune encephalitis, catatonia, diagnosis, treatment

## Abstract

Catatonia can be associated with a diverse range of conditions, including autoimmune encephalitis. Although rare, autoimmune encephalitis accounts for a significant proportion of catatonia cases with autoimmune aetiologies. In instances where autoimmune mechanisms are suspected, autoantibody testing is a key component of the diagnostic evaluation. However, test results should always be interpreted in conjunction with clinical findings. This article highlights the diagnostic challenges involved, advocating for structured diagnostic algorithms and timely initiation of immune therapy in carefully selected cases – particularly when antibody confirmation is absent. It revisits the paper, ‘Retrospective chart review of cases with steroid-responsive catatonia: exploring a potential autoimmune etiology’.

**Figure:**
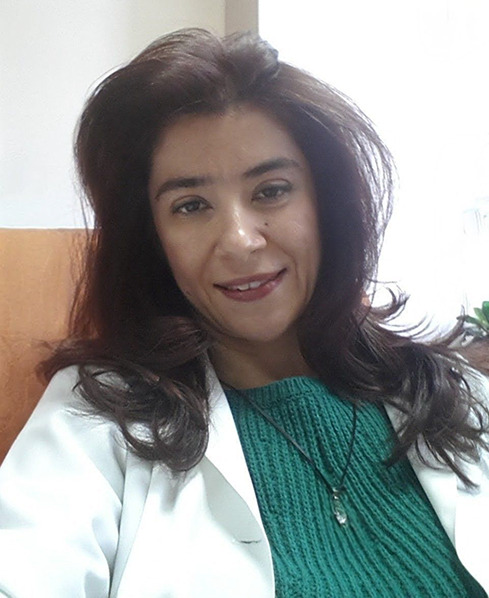

Historically described within the concept of schizophrenia, catatonia is a neuropsychiatric syndrome that can emerge due to a range of psychiatric disorders, general medical conditions and substance use.^1^ The International Statistical Classification of Diseases and Related Health Problems, version 11 (ICD-11) recognises catatonia as an independent disorder and describes four catatonia diagnoses: catatonia associated with another mental disorder, catatonia induced by substances or medications, secondary catatonia syndrome and catatonia, unspecified.^2^

A comprehensive understanding of the conditions associated with catatonia is the first step in addressing the potential causes of catatonia and implementing targeted therapeutic interventions. All patients experiencing an initial episode of catatonia should undergo a systematic evaluation guided by the evidence regarding the relative prevalence of underlying causes.^3^ This evaluation involves direct and collateral history-taking, observation, physical examination, relevant laboratory tests and additional investigations such as electroencephalography, neuroimaging, cerebrospinal fluid studies and antibody testing when necessary.^3,4^ It is widely suggested that the treatment of catatonia should address both the underlying condition and catatonia-specific symptoms simultaneously.^4^

In their recent paper, Ilhan et al retrospectively screened 56 patients hospitalised for subacute-onset catatonia in the psychiatry clinic of a university hospital, and presented a subset of 10 patients who had received empirical steroid treatment.^5^ Even though they did not have positive autoantibody markers or a confirmed diagnosis of autoimmune encephalitis, all ten cases met possible autoimmune encephalitis and/or probable anti-*N*-methyl-*D*-aspartate receptor (anti-NMDAR) encephalitis criteria^6^ (80% possible autoimmune encephalitis, 70% probable anti-NMDAR encephalitis), in addition to possible autoimmune psychosis criteria.^7^ Furthermore, all but 2 had antibody prevalence and an encephalopathy score (APE2)^8^ of 4 or above, indicating autoimmunity. Notably, all cases underwent a systematic, detailed assessment – including a comprehensive cerebrospinal fluid autoantibody test panel – prior to the initiation of steroid therapy, and eventually all demonstrated full symptomatic remission with steroids (methylprednisolone 1 g/day).

Autoimmune encephalitis is a rare but increasingly recognised disorder, with recent reports indicating a global increase in its prevalence.^9,10^ Clinically, it can present with a diverse range of symptoms that can overlap with other neurological and psychiatric conditions, including catatonia.^11^ Although it is an infrequent condition, autoimmune encephalitis represents approximately 75% of catatonia cases associated with autoimmune aetiologies, the vast majority of which are anti-NMDAR encephalitis.^12^

Autoimmune encephalitis diagnosis is based on clinical presentation, neuroimaging, cerebrospinal fluid analysis and identification of relevant autoantibodies, and often has a subacute onset.^11^ In the subacute-onset catatonia cohort screened by Ilhan et al, 17.9% (10 out of 56) of the patients had possible autoimmune encephalitis and/or probable anti-NMDAR encephalitis.^5^ All remaining 46 patients had a definitive diagnosis unassociated with autoimmune encephalitis or autoimmunity (except for one case with antiphospholipid antibody-associated vascular dementia).

There is evidence that approximately 6% of catatonia cases observed in general hospitals may be associated with encephalitis, including infections and paraneoplastic conditions along with autoimmune encephalitis.^13^ The high ratio of autoimmune encephalitis reported by Ilhan et al can be attributed to the selective inclusion of subacute-onset cases, multiple diagnostic criteria used and the rigorous diagnostic protocol for suspected autoimmunity adopted by the clinic. In addition, none of the diagnoses were definitive and the cohort represented patients from the adult psychiatry unit, not the general hospital population.

Just like overlooking cases, misdiagnosis of autoimmune encephalitis is also a concern because it may lead to unnecessary immune therapy and delayed appropriate interventions. A multicentre study revealed that autoimmune encephalitis misdiagnosis is common and might represent more than a quarter of cases diagnosed as autoimmune encephalitis in specialised clinics.^14^ Strikingly, it was reported that 72% of misdiagnosed cases did not fulfil diagnostic criteria for possible autoimmune encephalitis.^14^ By contrast, in Ilhan et al’s cohort, all patients fulfilled possible autoimmune encephalitis or probable anti-NMDAR encephalitis criteria along with possible autoimmune psychosis criteria. In addition, 80% had an APE2 score of 4 or above, indicating autoimmunity (validated for predicting neural autoantibodies or immunotherapy response in encephalopathy, APE2 can be used to guide diagnosis of seronegative autoimmune encephalitis employing specific clinical symptoms and history^8^).

Achieving an appropriate balance between preventing misdiagnosis and ensuring that autoimmune encephalitis cases are not missed is important. When catatonia is associated with autoimmune encephalitis, treatment should target both catatonia symptoms^4^ and involve immune therapies for autoimmune encephalitis.^11^ Benzodiazepines and electroconvulsive therapy (ECT) are typically the first options for managing catatonia symptoms.^4^ All patients presented by Ilhan et al were given lorazepam prior to methylprednisolone, but only 40% partially responded. When benzodiazepines and ECT are unsuccessful, it is essential to address the potential underlying causes of catatonia, such as autoimmune encephalitis, through appropriate treatment strategies.

First-line treatments for autoimmune encephalitis include corticosteroids,^11^ although their use can lead to serious side-effects such as infections, psychosis, avascular necrosis of the hip and heart failure, in addition to less severe but more frequent side-effects including insomnia, weight gain and irritability.^14^ Therefore a baseline screening – involving blood pressure, body mass index, blood glucose, triglycerides, electrolytes, urea, eye examination, bone mineral density and evaluation of pre-existing conditions – should be conducted beforehand.

Prompt use of immune therapies, such as steroids, in wisely selected patients can be a life-saver. While antibody confirmation remains crucial for a definitive diagnosis, syndrome-based identification of possible and probable cases of encephalitis is recommended to guide the timely initiation of therapy.^6^ As a matter of fact, evidence suggests that antibody test results should always be interpreted alongside clinical information.^10^

Ilhan et al’s small cohort represents a very good example of this fine balance, especially considering that all patients responded to steroid therapy despite negative autoantibody test results. It suggests that in-depth clinical and paraclinical evaluation of patients, and utilisation of multiple diagnostic criteria, increase the likelihood of identifying treatable cases while minimising the risk of misdiagnosis. This reinforces the importance of careful patient selection via clinical judgement and structured diagnostic algorithms, ensuring that treatable cases are not overlooked amid diagnostic uncertainty.

Catatonia is predominantly associated with psychiatric diagnoses,^13^ with psychiatrists most commonly encountering such cases. Similarly, autoimmune encephalitis frequently presents with psychiatric symptoms, highlighting the importance of psychiatric assessment and management in both the diagnosis and follow-up of autoimmune encephalitis cases.^11^ Robust collaboration among psychiatry, neurology and other disciplines of medicine, and extensive research to better identify the underlying neurobiological mechanisms of autoimmune encephalitis and catatonia, are critical in optimising patient outcomes.

Ilhan et al’s paper also reminds us of the importance of case reports and case series. Case reports can provide valuable clinical information often missed in large clinical studies, through direct observation and examination of individuals.^15^ In regard to rare conditions such as autoimmune encephalitis or catatonia, case series are helpful sources for building up the necessary knowledge and lay out the foundations for future research studies. Detailed case analyses and small cohort studies not only inform best practices but also help refine criteria for recognising treatable rare conditions that present with neuropsychiatric manifestations, such as autoimmune encephalitis.
